# Self-Referenced Refractive Index Biosensing with Graphene Fano Resonance Modes

**DOI:** 10.3390/bios11100400

**Published:** 2021-10-17

**Authors:** Xiaoyu Dai, Banxian Ruan, Yuanjiang Xiang

**Affiliations:** 1 College of Electrical and Information Engineering, Hunan University, Changsha 410082, China; xiaoyudai@hnu.edu.cn; 2 School of Physics and Electronics, Central South University, Changsha 410083, China; 2161190229@email.szu.edu.cn; 3 Scholar of Physics and Electronics, Hunan University, Changsha 410082, China

**Keywords:** biosensors, Fano resonance, graphene

## Abstract

A hybrid structure composed of periodic monolayer graphene nanoribbons and a dielectric multilayer structure was designed to generate a Fano resonance (FR). The strong interaction between the surface plasmon resonance of graphene and the dielectric waveguide mode results in the FR. The finite element method is utilized to investigate the behaviors of the FR, and it matches well with the theoretical calculations using rigorous coupled wave theory. The results demonstrate that the profile of the FR can be passively tuned by the period of the graphene nanoribbons and actively tuned by the Fermi level of the graphene. The decoupled nature of the FR gives it potential applications as a self-calibrated refractive index biosensor, and the sensitivity can reach as high as 4.615 μm/RIU. Thus, this work provides a new idea for an excellent self-referencing refractive index biosensor.

## 1. Introduction

The surface plasmon polariton (SPP), a surface evanescent wave formed by the interaction of free electrons and photons on the surface of a metal or an insulator, has been regarded as the information carrier in micro-nano optics with the most potential. As the SPP can break through the diffraction limit in traditional optics and support the propagation of light in acceptable wavelengths, it has promoted the development and manufacturing of optical devices [1,2]. In addition, the SPP is a non-radiation mode with a large near-field enhancement effect, giving it potential applications in photonic circuits [3], optical absorbers [4,5], optical sensors [6,7,8], and optical switches [9]. However, traditional metal-based plasmon devices suffer from high ohmic loss and poor active tunability, which restrict their practical applications.

Graphene, emerging as a promising plasmon material, has been studied intensively due to its excellent photoelectric properties, including low losses, tight field localization, broad response spectrum, and flexible tunability. The extraordinary plasma properties of graphene make it active in various coupling systems, such as plasmon-induced transparency [4,5] and Fano resonances (FRs) [10,11,12,13,14,15,16,17,18]. FRs are generally considered to be a feature of quantum systems and are also observed in optical systems. Generally, FRs are generated by the interaction between a narrow resonance and a broad resonance [19], and the sharp asymmetric spectra of FRs promise applications in nonlinear [20], lasing [21], switching [22], and sensors [23]. Much of the work on graphene-based FRs so far has focused on graphene-based metastructures or graphene-metal hybrid structures. While the intrinsic loss of graphene always causes a low-quality factor of the FR, it should be noted that an FR with a high-quality factor always indicates a high-performance device. In order to achieve a tunable FR with a high-quality factor, the coupling between the graphene surface plasmon resonances (SPRs) and the other resonant modes with high quality is an effective method.

In this work, we proposed a hybrid structure constructed by periodic monolayer graphene nanoribbons and a dielectric waveguide to realize an active tunable FR, where the waveguide mode supports the narrow resonance, and the graphene plasmon provides the broad resonance. An FR with a high-quality factor can be observed when the graphene SPR is coupled with the waveguide mode. The finite element method (FEM, Comsol Multiphysics) was utilized to simulate the behaviors of the FR. To confirm the accuracy of the results, the results were also calculated by rigorous coupled wave theory (RCWA) [24,25]. The results have shown that the profile of the proposed FR can be tuned passively by the period of graphene nanoribbons and can be tuned actively by the surface conductivity of the graphene. Meanwhile, it was found that the broad resonance of the proposed FR was very sensitive to the surrounding refractive index (RI), while the sharp resonance was insensitive. Therefore, the proposed FR satisfied the conditions for application to a self-reference sensor with the sharp resonance as the reference channel. Compared with the traditional single detect-signal optical sensors, which are easily affected by the unstable environment, the self-reference sensors are more accurate in the evaluation of the analyte. It is worth mentioning that based on the decoupling characteristics of the FRs, the sensitivity of the proposed self-reference RI sensor can be as high as 4.615 μm/RIU, which is significantly better than the sensors presented in Refs. [26,27,28]. Therefore, this work provides a new idea for an excellent self-referencing RI sensor.

## 2. Structure and Theoretical Model

**Figure 1:**
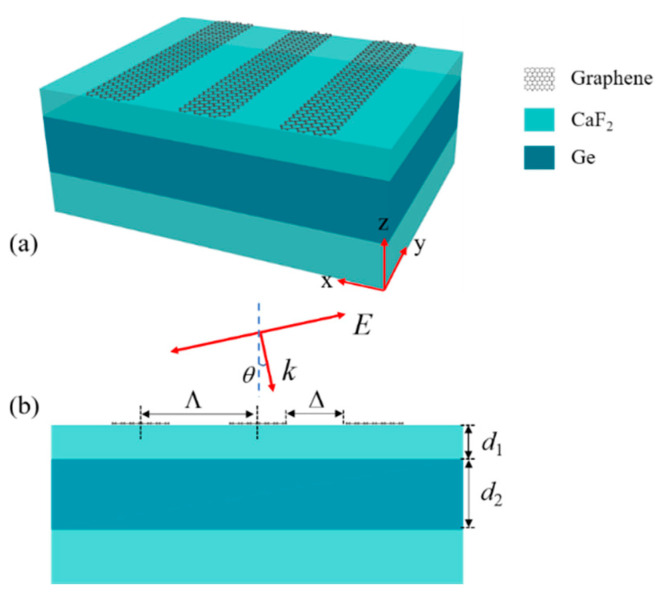
(**a**) Schematic diagram of designed structure. (**b**) Cross-sectional view of the structure in Figure 1a.

Figure 1a is a stereoscopic diagram of the proposed structure. The hybrid system consists of periodic monolayer graphene nanoribbons and a 3-layer planar waveguide (PWG) structure. Figure 1b shows a cross-sectional view of the structure and geometric parameter information. The graphene nanoribbons with period Λ and the groove length Δ in the x-axis direction are arranged on the PWG. In the following discussions, the substrate and the cladding layer of the PWG are considered to be CaF_2_, with the refractive index *n*_1_ = 1.3; the PWG core is set to be Ge with the refractive index *n*_2_ = 4. The thickness of each layer is set to be *d*_1_ = 1.2 μm and *d*_2_ = 2.8 μm, respectively. Then, the FR can be excited at the response wavelengths when the transverse magnetic (TM) light is incident on the structure with an incident angle *θ*. For the experimental fabrication of this structure, the dielectric multilayer can be prepared by the electron beam deposition method. The monolayer graphene can be grown by chemical vapor deposition (CVD) and transferred onto the planar waveguide. Then, the monolayer graphene can be utilized to fabricate the graphene nanoribbons by the atomic-force-microscope- (AFM) based lithography method [29].

**Figure 2:**
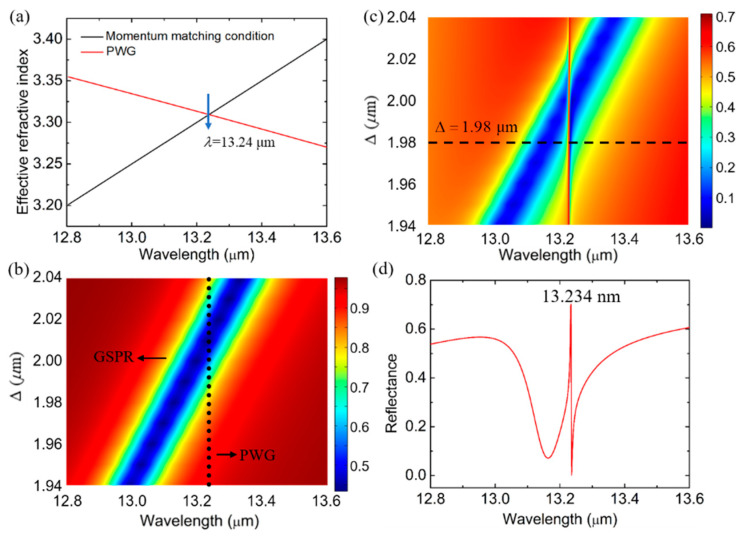
(**a**) Effective refractive index of PWG mode (red-line) and the momentum-matching condition supported by graphene grating (black-line). (**b**) The transmittance contour plot of wavelengths and Δ of the structure that is composed of periodic monolayer graphene nanoribbons and a CaF_2_ substrate. (**c**) The reflectance contour plot of wavelengths and Δ of the proposed structure. (**d**) The reflectance at Δ = 1.98 μm.

As is mentioned above, the FR is achieved by the coupling between a broad resonance and a narrow resonance. In this hybrid structure, the graphene nanoribbons were utilized to excite a surface plasmon resonance as the broad resonance. In Figure 2b, we plotted the transmission spectrum with the different Δ of the structure that was composed of the periodic monolayer graphene nanoribbons and a CaF2 substrate, and the graphene surface plasmon resonance (GSPR) could be observed clearly. The refractive index of monolayer graphene $n_{g}=\sqrt{1+i\sigma /(\omega \varepsilon_{0}d_{g})}$ was utilized for the simulation and theoretical calculations, where *ω* is the angular frequency, *ε*_0_ is the vacuum permittivity, *d*_g_ = 0.34 nm is the thickness of the monolayer graphene, and σ is the surface conductivity of the graphene constructed by intraband σ_intra_ and interband σ_inter_, described as [14]

(1) $$\sigma_{\mathrm{intra}}=i\frac{e^{2}k_{B}T}{\pi \hbar^{2}(\omega +i\tau )}[\frac{E_{F}}{k_{B}T}+2\ln(e^{-\frac{E_{F}}{k_{B}T}}+1)]$$

(2) $$\sigma_{\mathrm{inter}}=i\frac{e^{2}}{4\pi \hbar }\ln\left|\frac{2E_{F}-(\omega +i\tau )\hbar }{2E_{F}+(\omega +i\tau )\hbar }\right|$$

where *k_B_* is the Boltzmann constant, *ћ* is the reduced Planck constant, *T* expresses the absolute temperature of the environment, *e* is the elementary charge, and the carrier relaxation time *τ* is expressed as $\tau =\mu E_{F}/(ev_{f}^{2})$, depending on the Fermi velocity *v_f_*, the carrier mobility *μ*, and the Fermi energy *E_F_*. In this work, the Fermi velocity was set to be $v_{f}=10^{6}m/s$ and the carrier mobility was 1 m^2^/Vs.

The narrow resonance was provided by the waveguide mode in this coupled system. In order to make the waveguide mode couple with the GSPR, we calculated the dispersion of the PWG mode to obtain the resonance wavelength of the waveguide mode, and the dispersion is expressed as [30]

(3) $$\sqrt{n_{2}^{2}k_{0}^{2}-\beta^{2}}d_{2}=m\pi +2\arctan(\frac{n_{2}^{2}}{n_{1}^{2}}\sqrt{\frac{\beta^{2}-k_{0}^{2}n_{1}^{2}}{k_{0}^{2}n_{2}^{2}-\beta^{2}}})$$

where *k*_0_ is the wave vector of the plane incident wave in free space, *β* is the propagating constant, and *m* is the mode order of PWG. By solving equation (3) and *n*_eff_ = *β*/*k*_0_, the effective refractive index was obtained and was plotted as the red solid line in Figure 2a. In this hybrid structure, the momentum was provided by the graphene grating to excite the waveguide mode, and the momentum matching condition was expressed as *β* = *k*_0_sin*θ* ± 2*n*π/Λ. Hence, the theoretical resonant wavelength of the waveguide mode could be easily derived. With the incident angle *θ* = 0°, the period Λ = 4 μm, the waveguide mode order *m* = 0, and the diffraction mode order of grating *n* = 1, the resonant wavelength was calculated to be 13.24 μm, which was depicted in Figure 2a. From the formula of the momentum matching condition provided by the grating, we can know that the resonant wavelength of PWG is fixed at 13.24 with different Δ, as we plotted in Figure 2b (black solid dots). When the PWG was added to the graphene plasmon nano system, we simulated the reflectance spectrum with different Δ; in Figure 2c, the interference between the graphene plasmon and the waveguide mode is clearly illustrated. We can see that there is a wide low-reflection band generated by the graphene plasmons in Figure 2c, and the wide low-reflection band is cut off by an extremely sharp high-reflection band that is generated by the waveguide mode. The sharp resonance provided by the waveguide mode is fixed at 13.234, which is consistent with the theory that calculated the resonant wavelength at 13.24 μm, indicating the accuracy of our results. Finally, in order to intuitively observe the shape of the FR, we plotted the reflectance spectrum when Δ = 1.98 μm in Figure 2d. Obviously, it is a sharp asymmetrical line shape.

## 3. Results and Discussion

**Figure 3:**
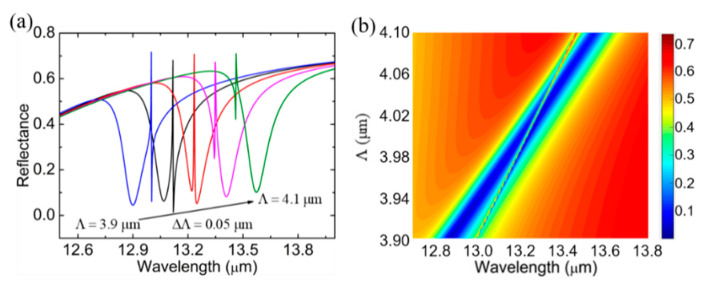
(**a**) The reflection spectrum of the structure when Λ changes from 3.9 to 4.1 μm with a step size of 0.05 μm. (**b**) The reflectance contour plot of wavelengths and Λ.

Figure 2b shows that the groove Δ plays a vital role on the shape of the FR. For a grating, the period Λ is a key parameter of the momentum-matching condition, and one may wonder how it influences the FR. So, in Figure 3, with Δ = 2 μm, we show the reflection spectra when the period Λ of the graphene nanoribbon is varied from 3.9 μm to 4.1 μm with a step size of 0.05 μm. As the spectrum depicts, an increase in Λ causes an overall redshift of the FR. The increased period of the graphene nanoribbon will cause a redshift of the graphene SPR. Besides, the changed period of the graphene grating will lead to a redshift of the waveguide mode resonance, which can be revealed by the dispersion relation of the PWG. In summary, the FR phenomenon is also redshifted overall. Combined with the continuously varying period sizes, the continuously varying FR response reflection spectra can be obtained in Figure 3b to more visually express the effect of Λ on the system response. Apparently, the resonance intersection of the graphene SPP and the PWG modes can be adjusted by changing the period of the graphene nanoribbons in the structure.

**Figure 4:**
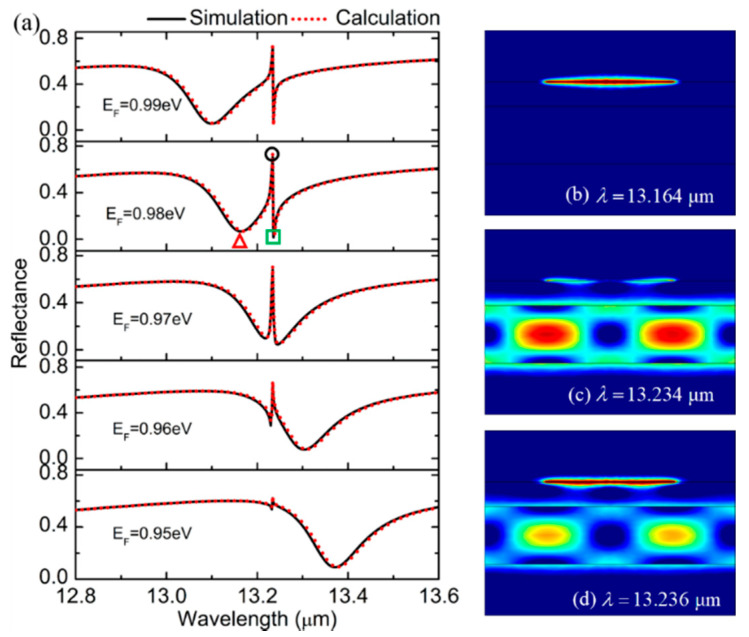
(**a**) Numerical simulation and theoretical calculation of reflectance spectra of the proposed structure when *E_F_* = 0.95, 0.96, 0.97, 0.98 and 0.99 eV. (**b**–**d**) The electric field distribution at the wavelengths marked by ‘△’, ‘○’ and ‘□’ in Figure 4a.

To investigate the dynamic tunability of the FR in the proposed structure, the Fermi energy *E_F_* of the graphene was tuned from 0.95 eV to 0.99 eV. The reflection spectra were simulated by the FEM (black solid line) and theoretically calculated by the RCWA (red dash line), as seen in Figure 4a. The consistency of the calculation results indicated the accuracy of our results. Obviously, when changing the Fermi level, the state of the waveguide mode will remain unchanged, and we can observe that the sharp resonance provided by the PWG mode is fixed. Hence, when the Fermi energy is changed, the change in the shape of the FR is mainly caused by the shift of the broad resonance supported by the graphene plasmon. The shape of the FR can be seen to transfer from asymmetric to symmetric and then to asymmetric in Figure 4a. To further investigate the origin of the FR, the electric field distributions were plotted as in Figure 4b–d, corresponding to the wavelength that is labeled as ‘△’, ‘○’ and ‘□’ in Figure 4a, respectively. It was found that the electric field was mainly concentrated on the graphene nanoribbons, as in Figure 4b, at the wavelength of *λ* = 13.164 μm, indicating the graphene SPP. As seen in Figure 4c,d, the electromagnetic energy was mainly confined at the graphene nanoribbons and planar waveguide, at the wavelengths of 13.234 μm and 13.236 μm, respectively, which demonstrated that both the waveguide mode and the graphene SPP were excited. Therefore, it indicates that the FR originates from the strong interaction between these two modes in the coupled structure.

**Figure 5:**
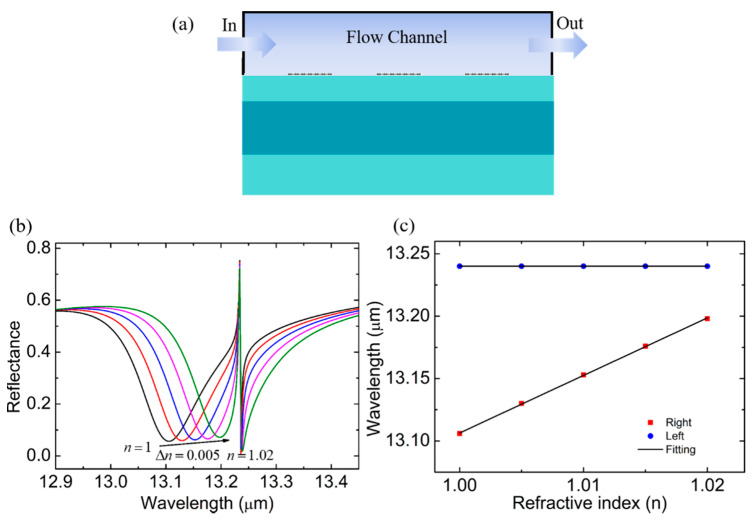
(**a**) Schematic diagram of the self-referencing biosensor structure. (**b**) The reflection spectrum when the RI of surrounding analyte varied from 1 to 1.02 with a step size of 0.005. (**c**) Relationship between the surrounding RI and the resonant wavelength according to Figure 5b.

According to the previous studies [10,12], FRs have a good application in RI sensors, and the behaviors of the proposed structure in RI sensing are explored in Figure 5. Changes in the analyte concentration caused a change in the refractive index. In this work, the analyte was considered to be a gas. Therefore, we plotted the reflection spectra at the different RI of the analyte *n* from 1 to 1.02, as shown in Figure 5a, to reveal the feasibility of the proposed structures in terms of the RI sensors. We observed that the left resonance was very sensitive to the variation of the surrounding *n*, while the wavelength of the right resonance was almost fixed. It promises a potential application for self-referencing RI sensors. In this sensing system, the right resonance works as a reference signal, and the absolute RI can be measured according to the calibration curve when the calibration samples are introduced. We plotted the change curve of the resonant wavelength versus *n* in Figure 5b and found that the FR position was insensible to the evolution of the solution/analyte RI. The graphene SPR mode position linearly changes with the RI, where the slope called sensitivity *S* = Δ*λ*/Δ*n*, can be as high as 4.615 μm/RIU, which is significantly better than the sensors presented in Refs. [26,27,28]. The excellent performance of the designed structure as a self-referencing RI sensor has an important potential value in applications, such as biomedical, environmental monitoring, and production safety.

## 4. Conclusions

In summary, we proposed an actively tuned FR constructed by periodic monolayer graphene nanoribbons hybridized on a distributed planar waveguide. In this structure, the periodic monolayer graphene nanoribbons can not only excite the graphene SPPs, but can also work as a grating to provide the momentum-matching condition for exciting the waveguide mode. The FR can be observed when these two modes are coupled with each other. The influences of the period and groove of the periodic monolayer graphene nanoribbons on the shape of the FR were discussed and it was found that the period and groove of the periodic monolayer graphene nanoribbons played a crucial role in the FR shape. In addition, the studies have shown that the shape of the FR can be tuned dynamically by the Fermi energy of graphene. Finally, we applied the results to the self-referenced RI sensor, and its sensitivity could reach 4.615 μm/RIU. Thus, this work provides a new idea for an excellent self-referencing RI sensor.

## Data Availability

The data presented in this study are available on request from the corresponding author.
